# Electroanalytical Overview: The Sensing of Mesalamine (5-Aminosalicylic Acid)

**DOI:** 10.1021/acsmeasuresciau.3c00061

**Published:** 2023-12-12

**Authors:** Robert D. Crapnell, Prashanth S. Adarakatti, Craig E. Banks

**Affiliations:** Faculty of Science and Engineering, Manchester Metropolitan University, Chester Street, Manchester M1 5GD, United Kingdom

**Keywords:** 5-aminosalicylic acid, mesalamine, electroanalytical, sensors, electrochemistry, electroanalysis, inflammatory bowel disease treatment, Crohn’s disease

## Abstract

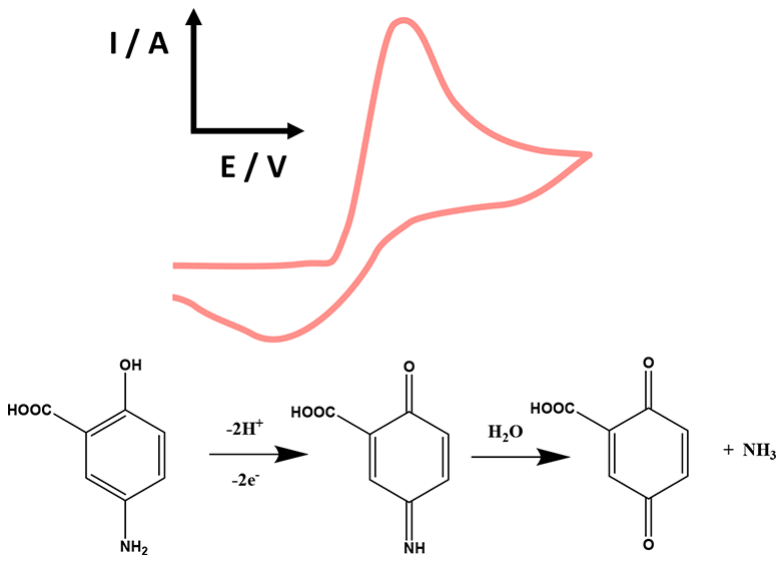

Mesalamine, known as 5-aminosalicylic acid, is a medication used primarily in the treatment of inflammatory bowel disease, including ulcerative colitis and Crohn’s disease. 5-Aminosalicylic acid can be measured using various benchtop laboratory techniques which involve liquid chromatography–mass spectroscopy, but these are sophisticated and large, meaning that they cannot be used on-site because transportation of the samples, chemicals, and physical and biological reactions can potentially occur, which can affect the sample’s composition and potentially result in inaccurate results. An alternative approach is the use of electrochemical based sensing platforms which has the advantages of portability, cost-efficiency, facile miniaturization, and rapid analysis while nonetheless providing sensitivity and selectivity. We provide an overview of the use of the electroanalytical techniques for the sensing of 5-aminosalicylic acid and compare them to other laboratory-based measurements. The applications, challenges faced, and future opportunities for electroanalytical based sensing platforms are presented in this review.

## 1 Introduction

**Scheme 1:**
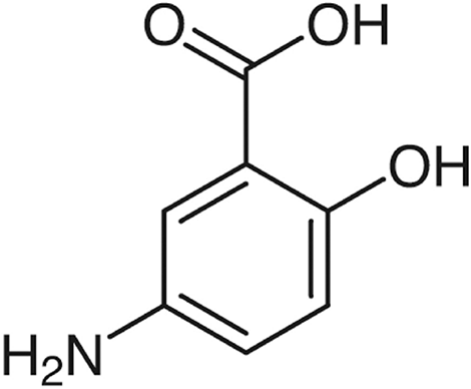
Molecular Structure of 5-Aminosalicylic Acid

5-Aminosalicylic acid (mesalamine/mesalazine) has been used as a nonsteroidal anti-inflammatory drug to treat inflammatory bowel disease, including ulcerative colitis, inflamed anus, and Crohn’s disease, and also it may have antineoplastic and potentially prophylactic chemopreventive properties;^1−5^ see Scheme 1 for the molecular structure of 5-aminosalicylic acid.

5-Aminosalicylic acid (5-ASA) can be synthesized via a Kolbe reaction which consists of reacting *m*-amino-phenol with potassium bicarbonate and carbon dioxide while heating at pressures of 5 to 10 atm.^6^ 5-Aminosalicylic acid is known to inhibit the production of cytokines and inflammatory mediators but the underlying mechanism of the intestinal effects of 5-ASA remains unidentified.^7^ Once 5-ASA is taken, it is metabolized to its derivative *N*-acetylated-5-ASA by *N*-acetyltransferase in the intestinal tract and liver where the *N*-acetylated-5-ASA is an antioxidant that attracts free radicals which is hypothetically injured by the creation of metabolism.^8^

5-ASA is required to be analytically measured related to its therapeutic use in the treatment of inflammatory bowel disease where levels in the blood or plasma allow the concentration of the medication in a patient’s body to be known, ensuring that it falls within the therapeutic range. Monitoring 5-ASA levels can help optimize the dosing regimen, ensuring that the drug is effective and not being underdosed or overdosed. Through the monitoring of 5-aminosalicylic acid levels, healthcare providers can assess the efficacy of the medication in controlling inflammation in the gastrointestinal tract, where if 5-ASA levels are consistently low, despite adherence to the treatment, it may suggest that the patient requires a higher dose or that alternative treatment options need to be considered. Some patients with inflammatory bowel disease may not respond to 5-ASA treatment due to inherent resistance or individual differences in drug metabolism. Clearly there is a need for the analytical detection of 5-ASA.

There are of course many laboratory based systems that can measure 5-ASA, including spectrophotometry,^9^ high-performance liquid chromatography with fluorescence,^10^ proton nuclear magnetic resonance spectroscopy,^11^ liquid chromatography with positive-ion electrospray ionization mass spectrometry,^12^ electrospray ionization tandem mass spectrometry,^13^ and quadrupole time-of-flight mass spectrometry,^14^ while noting that using liquid chromatography methods makes the analytical run time per sample take from 10 to 40 min. In addition, they are costly, sophisticated, and the instruments are large, meaning that they are not able to be applied for use in on-site determination.^15^ In the transportation of the sample from clinic or hospital, chemical, physical, and biological reactions can potentially occur which can affect the sample’s composition and potentially result in inaccurate results. The development of new in situ sensors is needed to reduce the time, cost, and sampling of such monitoring studies in order to mitigate these problems. One response to providing in situ sensors is electroanalytical based, which can provide selectivity, sensitivity, cost efficiency, facile miniaturization, minimal sample preparation, low-cost instrumentation, and rapid analysis that can be deployed on site, such as with healthcare providers. A review has been published^16^ which considers the different analytical and bioanalytical methods for the quantification of 5-ASA but it is lacking emphasis on electroanalytical sensing platforms, which we provide herein. Consequently, we overview the recent approaches to the development of electroanalytical based sensors and provide future research directions.

## 2 Electroanalytical Sensing of 5-Aminosalicylic Acid (5-ASA)

Table 1 summarizes the recent approaches to the electroanalytical sensing of 5-ASA, which are grouped by the underlying working electrode, and compares the modification of the working electrode surface, linear ranges, limits of detection (LoD), and use in the sensing of 5-ASA within real samples. We first highlight the most beneficial electroanalytical sensing platforms for 5-ASA starting with glassy carbon electrodes.

**Table 1:** Overview of the Electroanalytical Literature Directed to the Sensing of 5-ASA^a^

electrode material	electrode modification	electroanalytical technique	dynamic range	limit of detection	real sample composition	reference
GCE	polypyrrole	LSV	0.01–0.1 μM	3 nM	human plasma and pharmaceutical	(32)
GCE	SrSnO_3_	DPV	0.01–212 μM	2 nM	human urine, lake water and pharmaceutical	(34)
GCE	Co_2_SnO_4_/rGO	DPV	0.029–5.78, 15.69–1326 μM	4.9 nM	human urine, serum, river water and pharmaceutical	(36)
GCE		DPV	2–100 μM	0.816 μM	pharmaceutical	(27)
GCE	CD/HDCMAB/CHIT	Amperometry	0.1–10 μM	0.05 μM	human serum	(55)
GCE	WS_2_/rGO	DPV	0–300 μM	3 nM	tap water human serum and urine	(35)
GCE	CuW nanosheets	amperometry	0.005–367 μM	1.2 nM	human urine	(37)
GCE	FSO/SnO_2_/g-C_3_N_4_	DPV	0.05–1749 μM	7.5 nM	human urine and pharmaceutical	(63)
GCE	Ni-ZrO_2_/MWCNTs.	DPV	1 nM–500 μM	2.9 nM	human serum, urine and pharmaceutical	(33)
GCE	Ag dendrites/MIP	ASSWV	0.05–100 μM	15 nM	human urine and serum	(29)
GCE	poly(glutamic acid)	DPV	5 μM–0.5 mM	23.94 nM		(28)
GCE	power ultrasound	CV, SWV, sono LSV	20–180; 11–88; 1–57 μM	5; 3.6; 0.3 μM	tissue culture medium	(23)
GCE	Bi-EDTA	amperometry	0.005–585 μM	0.55 nM	human urine	(64)
GCE	molecularly imprinted sol–gel	DPV	2–20 μM	0.97 μM	pharmaceutical	(65)
GCE	poly(methylene blue)/CNT	DPV	5–100 μM	7.7 nM	pharmaceutical	(39)
GCE	poly(methylene blue)/Au NPs	amperometry	1–150 μM	64 nM	pharmaceutical	(38)
GCE	poly(murexide)	CV	250–1250 μM	91 μM	pharmaceutical	(66)
GCE	CNTs/Nafion	SWV	50 nM–2.5 μM	12 nM	human serum	(15)
GCE	CNT-NH_2_/CHIT	SWV	0.13–8 μM	0.21 μM	pharmaceutical and human serum	(41)
SPCE	CAs/Pd–WO_3_	amperometry	3 nM–350 μM	0.8 nM	human urine and blood serum	(46)
SPCE	g-C_3_N_4_/CeVO_4_	DPV	2 nM - 380 μM	5.47 nM	human blood serum and urine, river water and a pharmaceutical tablet	(45)
SPCE	ZnCr-LDH/WC	DPV	0.03–254 μM	6 nM	human urine and river water	(44)
pencil graphite	ds-DNA/PPy/SL-C/La^3+^-doped CuO	DPV	0.03–100 μM	9 nM	human urine, serum and pharmaceutical tablet	(60)
pencil graphite		SWV	0.978–72.5 μM	0.02 μM	pharmaceutical tablet	(58)
BDDE		SWV	2 μM–0.3 mM	0.7 μM	human urine and pharmaceutical tablet	(48)
CPE	polymerized-congo red	CV	80–200 μM	0.112 μM	pharmaceutical tablets	(57)
CPE	ZIF-67	DPV	0.03–50 μM	0.01 μM	human serum and urine	(56)
CPE	Co/Cu-BTC@SiO_2_ nanostructure	DPV	0.05–200 μM	100 nM	human plasma	(67)
CPE	sodium dodecyl sulfate	DPV	1–7 μM	0.238 μM	pharmaceutical tablet	(53)
CPE	cetyltrimethyl ammonium bromide	CV	60–140 μM	1.8 nM	pharmaceutical tablet	(54)
GPE	poly(benzoquinone) chromium(III) complex	DPV	2–600 μM	70 nM	pharmaceutical tablets	(52)

a **Key:** DPV: differential pulse voltammetry; GC: glassy carbon electrode; CV: cyclic voltammetry; SWV: square-wave voltammetry; LSV: linear sweep voltammetry; CA: carbon aerogels; PPy: polypyrole; SL-C: sponge like carbon; ZnCr-LDH: 2D ZnCr layered double hydroxide; WC: tungsten carbide composite; CPE: carbon paste electrode; GPE: graphite paste electrode; BTC: bimetallic; ZIF: Zeolitic imidazole frameworks; BDDE: boron-doped diamond electrode; MIP: molecular imprinted polymer; ASSWV: Anodic stripping square wave voltammograms; CNT: carbon nanotubes; Au NPs: gold nanoparticles; HDCMAB: hexadecyltrimethylammonium bromide surfactant; CHIT: chitosan; CD: carbon dots; rGO:reduced graphene oxide; and FSO: Fe_1.874_Sn_0.096_O_3_.

## 3 Glassy Carbon Electrodes (GCE)

**Figure 1:**
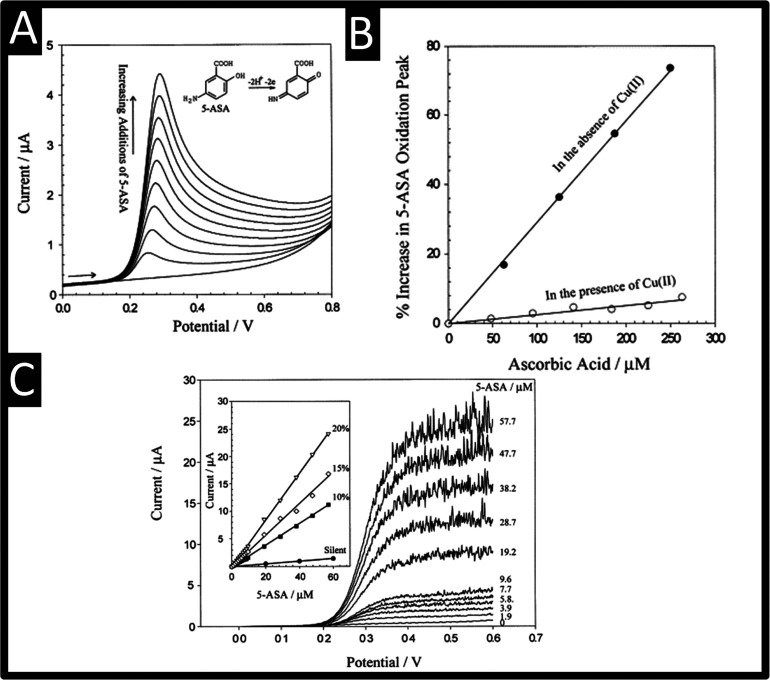
(A) Linear sweep voltammograms showing the electrochemical oxidation of 5-ASA, 0–180 μM; (B) the effect of ascorbic acid on the magnitude of the 5-ASA oxidation peak (20 μM) in the presence and absence of 0.5 mM cupric ion; (C) Linear sweep voltammograms showing the sonochemically enhanced oxidation of 5-ASA (0–57 μM), while the inset shows the influence of the ultrasound intensity on the magnitude of the limiting current. Parameters: glassy carbon electrode; scan rate: 50 mV s^–1^; pH 7. Reproduced from ref (23). Copyright 2001 Elsevier.

Glassy carbon electrode (GCE) is a well-utilized electrochemical surface due to its high temperature resistance, hardness, low density, low electrical resistance, good electrical conductivity, and wide potential window.^17−20^ One downside is that the electrode needs to be polished each time using alumina sprays of various grades, which can contribute to its poor reproducibility. The electrochemical oxidation of 5-ASA has been reported as the basis of post column detection systems using high-performance liquid chromatography with a glassy carbon electrode used in its determination within serum^21^ and within intestinal endoscopic biopsy samples.^22^ As shown in Figure 1A, the electrochemical oxidation of 5-ASA gives rise to well-defined signals using a glassy carbon electrode which occurs at +0.2 V (vs. SCE), where the mechanism occurs via a proton-coupled electron transfer process involving 2 protons and 2 electrons into a quinone-imine intermediate; this sensor allows the sensing of 5-ASA over the range of 20–180 μM with a reported LoD of 5 μM. Interestingly, the authors studied ascorbic acid which masks the signal of the 5-ASA. This is overcome through the use of cupric ions (∼0.5 mM) which scavenge the ascorbic acid resulting in the signal of the 5-ASA being “unmasked”; see Figure 1B.

Ultrasonically enhanced electroanalytical measurements have been successfully applied for the detection of a wide range of target analytes where the beneficial effects of power ultrasound, which has a frequency of 20 kHz, to electroanalysis allows the possibility for quantitative analysis in otherwise highly passivating media, where classical electrochemical techniques often fail, and provides cavitation processes with extra mass transport allowing low detection limits and enhanced sensitivity.^24,25^ To this end, the authors explored the use of power ultrasound during their sensing of 5-ASA, which is shown in Figure 1C, a characteristic limiting current behavior is observed with the spikes in the current attributed to cavitation processes at or near the vicinity of the electrode surface. Also shown is the inset in Figure 1C which shows the effect of silent, i.e., no power ultrasound, where the signal is elevated using 10, 15 and 20% amplitude. The use of the improved mass transport resulted in a linear range and LoD of 1–57 and 0.3 μM, respectively. Lastly, the authors demonstrated their sensing approach to the determination of 5-ASA within a tissue culture medium, resulting in a recovery of 102% suggesting that this method has efficacy.

**Figure 2:**
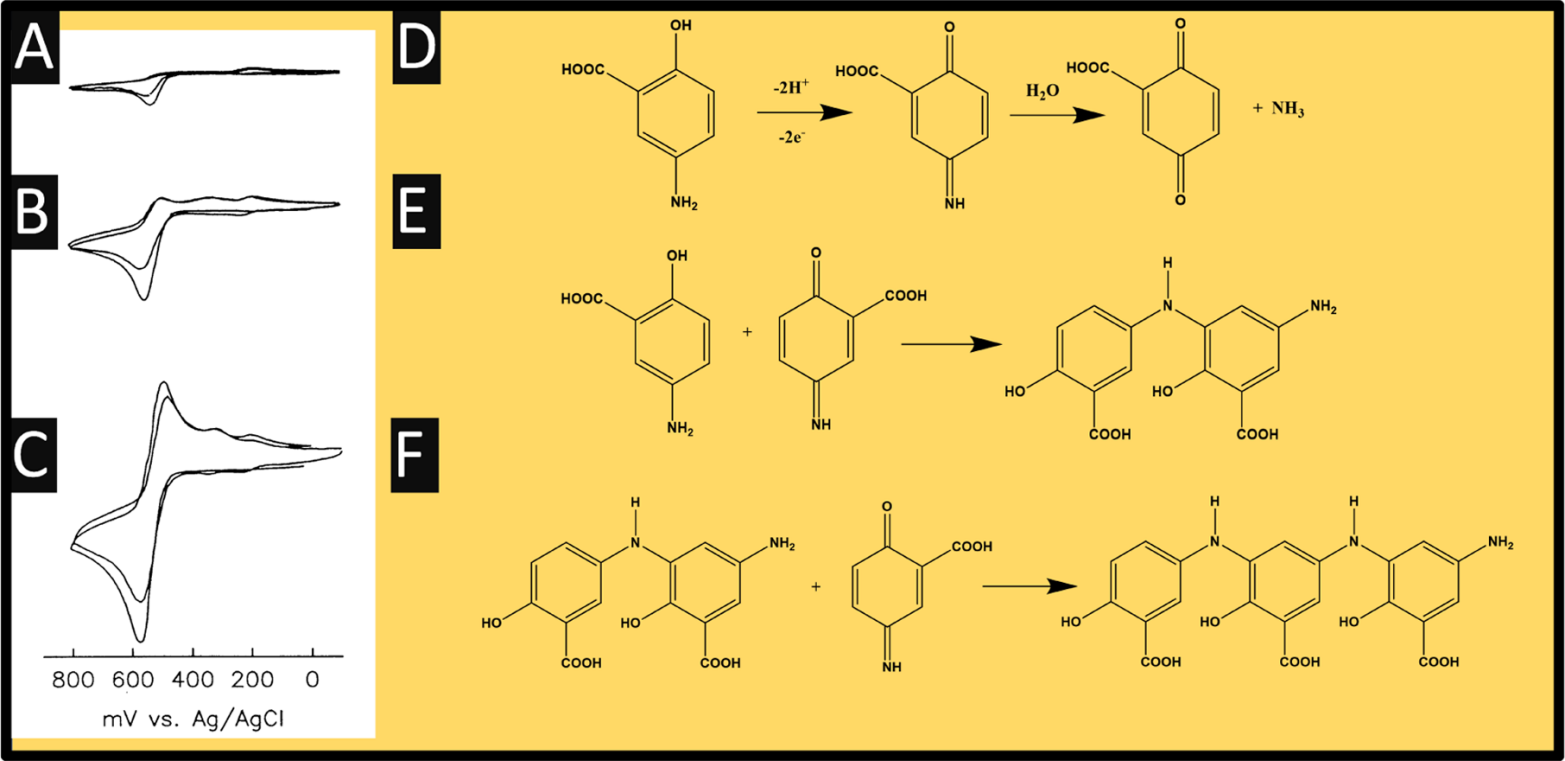
Cyclic voltammograms of 5-ASA (pH 2) recorded using a GCE at 10 (A), 100 (B), and 500 (C) mVs^–1^; also shown is the mechanism for the electrochemical oxidation of 5-ASA (D–F). Reproduced from ref (26). Copyright 1992 Springer Link.

The electrochemical oxidation mechanism of 5-ASA has been exemplified by Palsmeier et al.,^26^ who studied the degradation of 5-ASA using reverse-phase liquid chromatography combined with a photodiode array detection and using a dual-electrode parallel configuration while using mass spectroscopy to identify the products. As shown in Figure 2, the electrochemical oxidation of 5-ASA recorded at a GCE is shown for various scan rates.

At slow scan rates an irreversible oxidation peak is observed, while with increasing the scan rates the processes become quasi-reversible as can be seen with the presence of the reduction peak. The cyclic voltammetric profiles confirm that the mechanism is operating as in Figure 2 D, where it occurs via a proton-coupled electron transfer process involving 2 protons and 2 electrons into a quinone-imine intermediate, where at slow scan rates, the quinone-imine intermediate decomposes into gentisic acid, indicating that the electrochemical reduction cannot take place. When faster scan rates are used, the processes becomes quasi-reversible as shown in Figure 2C, where the electrochemical reduction can occur. Also shown are the products of the electrochemical oxidation of 5-ASA, which occurs over many hours where one pathway is a 1,4 Michael addition of excess 5-ASA to the 5-ASA quinonimine resulting in a reduced dimer, which can react further in the presence of additional quinonimine.^26^ Note that the p*K*_a_ needs to be considered, for example the primary aromatic amino group has a p*K*_a_ of 6, while the carboxylic group has a p*K*_a_ of 3, and the phenolic group’s p*K*_a_ is 13.9 in the 5-ASA molecule. As such the 5-ASA was completely in cationic form in the experimental conditions used.

Returning to the sensing of 5-ASA using a GCE, the measurement has been reported using a bare/unmodified GCE toward 5-ASA which reports a linear range of 2–100 μM with a LoD of 0.816 μM and was demonstrated to measure 5-ASA within a pharmaceutical tablet which was indirectly compared with HPLC.^27^ Noting the comparability to the analytical outputs reported using sonoelectroanalysis,^23^ one can question the use of using power ultrasound in the first place. Other work has reported the use of a GCE electrode modified by poly(glutamic acid), where they electropolymerized glutamic acid by sweeping the potential window from −1.0 to +1.3 V (vs. SCE) for 12 cycles at 100 mVs^–1^, which has a low LoD of 23.94 nM, but it is very limited as no real sample has been tested.^28^ Another approach has been reported by Torkashvand et al.^29^ using a molecularly imprinted polymer (MIP) casted upon a GCE, which gave rise to a linear range of 0.05–100 μM with a LoD of 15 nM. Using a MIP allows one to devolve a selective method toward 5-ASA that involves placing functional monomer around the 5-ASA target by covalent or noncovalent interaction followed by polymerization, after which the 5-ASA is removed providing a selective binding medium for the 5-ASA target.^30,31^ The authors used electrochemical polymerization using a monomer mixture of *O*-phenylenediamine and *p*-aminobenzoic acid. This MIP was modified with silver dendrites which have nanoscaled branches. The selectivity of the approach was determined against 5-ASA in the presence of minoxidil, warfarin, and phenylephrine which showed no response, indicating that the imprinted membranes have special recognition toward 5-ASA.^29^ The authors demonstrated their MIP was able to measure 5-ASA within human serum and urine, noting that in the former, serum samples are collected and treated with 1 mL methanol as serum protein precipitating agent, followed by centrifugation where the clear supernatant layer was filtrated through a milli-pore filter to produce a protein-free human serum. While in the case of the latter, urine was centrifuged and diluted with distilled water without any further pretreatment. These real samples were spiked with 5-ASA at the low micromolar level where recoveries were in the range of 98–102%, with a low RSD% (1.7–3.1).^29^ Related to MIPs is the use of nanoporous thin films of multiwalled carbon nanotubes deposited upon a GCE, where polypyrrole is formed via electrochemical polymerization which gave a linear range of 0.01–0.1 μM and a LoD of 3 nM.^32^ The authors reported that they can measure 5-ASA in the presence of azathioprine, as this becomes an interference to those who are under the risk of bone marrow depression,^32^ within model solutions (pH 2). However, this is pointless as they do not show this to be any real use in avoiding the determination of 5-ASA in the presence of azathioprine within real samples.

**Figure 3:**
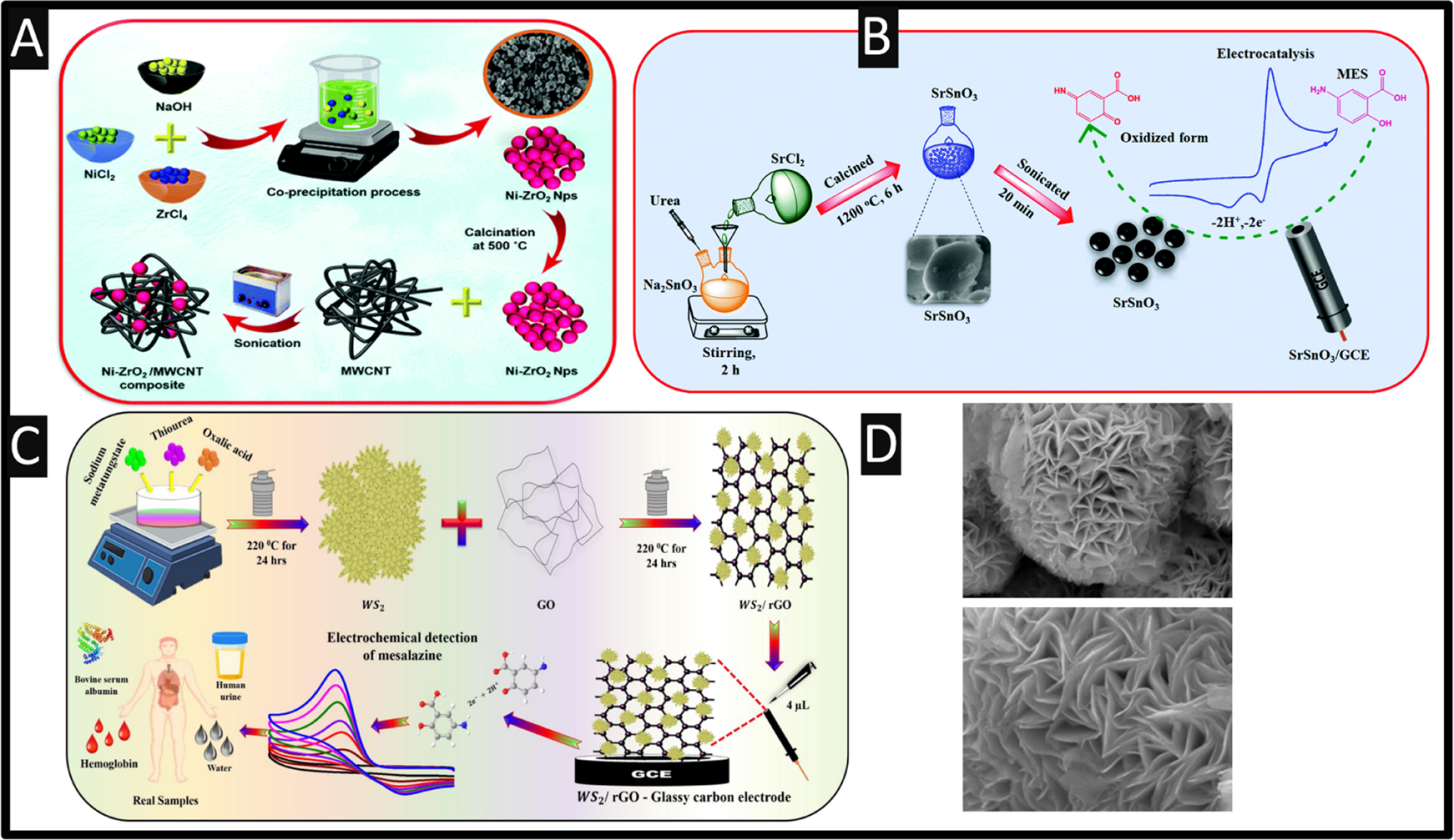
(A) An overview of the synthesis to obtain Ni-ZrO_2_/MWCNTs. Figure reproduced from ref (33). Copyright 2021 The Royal Society of Chemistry; (B) An illustration of SrSnO_3_ is fabricated and applied to the sensing of 5-ASA. Reproduced from ref (34). Copyright 2019 The Royal Society of Chemistry. (C) A summary of how they fabricated their WS_2_/rGO sensor. (D) Field emission scanning electron microscopy of the WS_2_. Reproduced from ref (35). Copyright 2019 Elsevier.

Other notable work has reported upon the use of Ni-doped ZrO_2_ nanoparticles supported upon multiwalled carbon nanotubes (MWCNTs) as the basis of a sensor that reports a low dynamic range 1 nM to 500 μM with a LoD of 2.9 nM. As shown within Figure 3A, the Ni-ZrO_2_ are fabricated via a coprecipitation methodology, where zirconium salt is dispersed into water with nickel chloride and sodium hydroxide being mixed for 4 h. This forms Ni-ZrO_2_ which is collected and calcined at 500 °C for 2 h, where the ratio between the Ni and ZrO_2_ is 2:1 with an average nanoparticle size of 100–150 nm. These nanoparticles were dispersed onto MWCNTs by mixing via ultrasonication. Using electrochemical impedance spectroscopy, the authors compared the charge transfer resistance (*R*_ct_) which is significantly lower, 23.8 Ω, compared to a bare GCE, 266.5 Ω, while in the case of Ni-ZrO_2_/GCE, 59.7 Ω and MWCNTs/GCE, 138.6 Ω which is attributed to the higher electrical conductivity through the use of ZrO_2_ with Ni combined with MWCNTs giving rise to a large surface electrochemical area.^33^ The authors demonstrated future use of their sensor by measuring spiked 5-ASA within human serum, urine, and a pharmaceutical, noting that human blood serum and urine samples were diluted in 25 mL with buffer, spiked with 5-ASA and are ready to be analyzed. The recoveries of 5-ASA from human blood, urine, and pharmaceutical varied from 95.92% to 99.92% suggesting that the proposed sensor has potential for use in practical applications in clinical samples.^33^ Also summarized within Figure 3B is how Muthukutty and co-workers^34^ synthesized a new perovskite-type sphere-like strontium stannate (SrSnO3) material by a simple coprecipitation methodology. Their approach used urea and salts of strontium and stannate, which are calcined at 1200 °C for 6 h resulting in the formation of SrSnO_3_, producing a particle size of 200 nm. The nanoparticles are “electrically wired” through depositing these onto a GCE which reported a range of 0.01–212 μM with a 2 nM LoD. The sensor was evaluated with the measurement of 5-ASA within pharmaceuticals, human urine, and lake water samples; simply, samples are collected and then centrifuged for 30 min, filtered via Whatman No. 1 filter papers and further diluted with 0.05 M PBS. They are then spiked with 5-ASA, with reported recoveries of 93.5–99.8%, showing the potential for its use to be taken up for the on-site measurement of 5-ASA but further research should be completed comparing this to a gold standard laboratory approach, e*.*g., liquid chromatography–mass spectrometry

Other work has been inspired by the use of SrSnO_3_ where self-assembled (1D) Co_2_SnO_4_ nanocubes were formed via hydrothermal synthesis and then integrated with reduced graphene oxide (rGO).^36^ This sensor was able to measure across the range of 0.029–1326 μM with a LoD of 4.9 nM, which was successful in the measurement of 5-ASA within spiked human urine, serum, river water, and pharmaceutical products. The use of the Co_2_SnO_4_/rGO nanohybrids displays excellent analytical results which are attributed to easy diffusion of the 5-ASA into the electrolyte and stronger interactions with the inner region of the Co_2_SnO_4_/rGO nanosurface, due to a reduction in intersheet aggregation where the presence of structural defects at reduced graphene oxide has efficiently promoted higher active sites for efficient electron transfer combined with Co_2_SnO_4_.^36^ Keerthana and co-workers use rGO decorated with WS_2_.^35^ As shown within Figure 3C, the WS_2_ was fabricated by using a tungsten salt, thiourea, and oxalic acid dissolved into water, which were then autoclaved at 220 °C for 24 h. This was then added to graphene oxide and autoclaved again at 220 °C for 24 h which produces a unique morphology, as shown in Figure 3D. Hierarchical flowers are shown, which through a combination of WS_2_ and rGO increase the surface area and the number of active sites, resulting in a unique material. The WS_2_/rGO is then drop-cast upon a GCE which shows a linear range of 0–300 μM toward 5-ASA and a LoD of 3 nM. The authors studied interferents at 10-fold higher than 5-ASA, namely: catechol, dopamine, sodium, glucose, potassium, hydroquinone, potassium bromide, sodium nitrite, and amino phenylacetate, which showed no response indicating a selective approach to the sensing of 5-ASA. The sensor was evaluated into the measurement of spiked 5-ASA within tap water, human serum, and urine which showed excellent recoveries of 97–102%. Copper tungstate nanosheets have also been developed to measure 5-ASA.^37^ In the synthesis of copper tungstate nanosheets, the authors combined sodium tungstate with copper chloride, which was placed into an ultrasonic bath for 1 h, after which the products were dried at 80 °C for 12 h. These generated copper tungstate nanosheets which had an average thickness of 50 nm, and was shown to sense 5-ASA over the range of 0.005–367 μM with a LoD of 1.2 nM. The authors demonstrated their sensor has selectivity toward 5-ASA, reporting that there is no change in the current response for 20-fold concentrations of azathioprine, ascorbic acid, 2-aminophen, potassium, sodium, tryptophan, dopamine, gallic acid, catechol, glucose, acetaminophen, salicylic acid, and tyrosine. The authors showed that their sensor has the potential to be used in the measurement of 5-ASA within spiked human urine reporting recoveries in the range of 95.0–99.2%, where a low% RSD is demonstrated (3.6–4.1).^37^

Other work has reported the use of a GCE composite composed of the electroactive redox polymer formed in a ternary deep eutectic solvents using gold nanoparticles^38^ or with carbon nanotubes.^39^ In the former case, they performed the simultaneous sensing of ascorbic acid and 5-ASA while in the latter, simultaneously acetaminophen and 5-ASA, both approaches showing low detection limits which are successful for determining 5-ASA within pharmaceuticals. Deep eutectic solvents are attracting attention since they are composed of at least one hydrogen bond donor and a hydrogen bond acceptor. They are classed as nontoxic green solvents, with properties which overcome the hazards of ionic liquids such as high toxicity, nonbiodegradability, and high costs.^40^ Relating to those simultaneous detections of 5-ASA, folic acid has also been reported using carbon nanotubes functionalized with amino groups coated with chitosan. The voltammetric peaks are well resolved with 5-ASA and folic acid reported at +0.33 and +0.81 V.^41^ This senor gave a linear range of 0.13–8 μM with a LoD of 0.21 μM which was shown to be successful for the determination in human serum samples; the output of the sensor and excellent electroanalytical performance are attributed to the strong adsorptive ability of ionized analytes and subtle electronic properties.^41^

## 4 Screen-Printed Carbon Electrodes (SPCEs)

**Figure 4:**
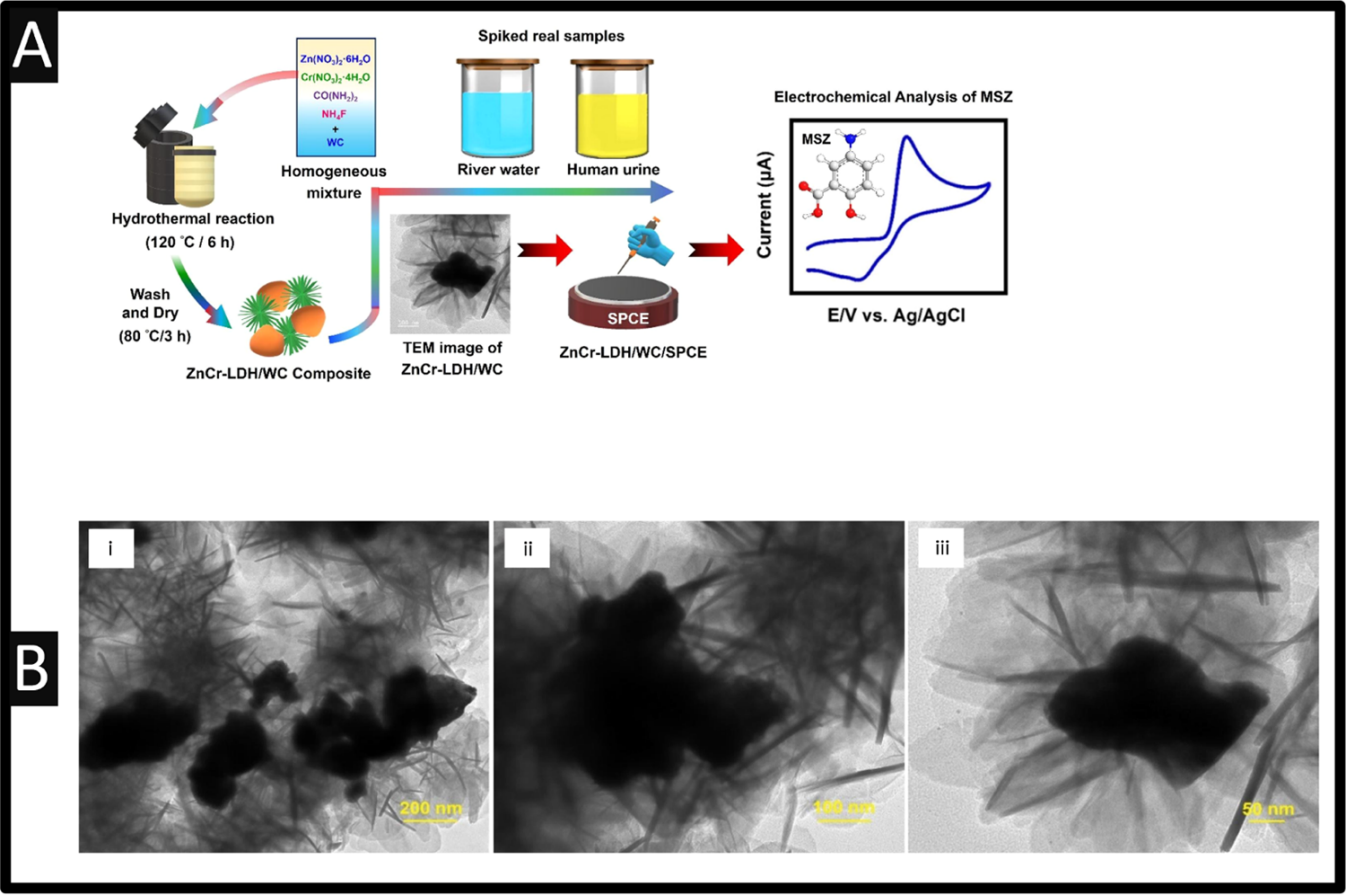
(A) Schematic diagram of the synthesis process of ZnCr-LDH/WC composite and its electrochemical applications; (B) TEM images of the ZnCr-LDH/WC composite. Reproduced from ref (44). Copyright 2023 Elsevier.

A notable approach is the use of screen-printed carbon electrodes, which allow different shapes and geometries to be realized, can be made of different materials, and can be suitably modified with a variety of biological elements and provide miniaturization helping for laboratory-based electroanalytical systems to be deployed within the field.^42,43^ Kokulnathan and co-workers have reported upon the hydrothermal synthesis of a 2D ZnCr layered double hydroxide (LDH)/tungsten carbide (WC) composite.^44^ As summarized within Figure 4A, the ZnCr-LDH was synthesized by the hydrothermal approach where salts of zinc and chromium were dissolved into deionized water at a stoichiometric ratio of 3:1 under constant magnetic stirring for 15 min. Next, the addition of tungsten carbide with ammonium fluoride and urea were dissolved into deionized water which is slowly poured into the zinc and chromium solution. This was mixed for 30 min and then placed into a Teflon-coated autoclave in an air-oven at 120 °C for 6 h. Then, the as-obtained precipitate was washed with water and ethanol to remove residuals and adsorbed impurities followed by drying under vacuum at 60 °C for 4 h. This ZnCr-LDH/WC composite had an irregular 2D flake-like morphology with an average size range of 100–300 nm akin to nanoflowers giving rise to improved electrochemical area; see Figure 4B TEM imaging of the composite at increasing magnifications (i–iii). The ZnCr-LDH/WC composite was drop-cast upon a SPCE and was found to exhibit a linear response of 0.03–254 μM toward 5-ASA with a LoD of 6.0 nM. The authors noted that the abundant surface-active sites, abundant synergistic sites, excellent conductivity, and fast charge carrier transport in the ZnCr-LDH/WC composite can effectively improve the electrochemical activity.^44^ The sensor was shown to successfully measure 5-ASA within spiked human urine and river water samples with recoveries of 96.80–99.60%.

**Figure 5:**
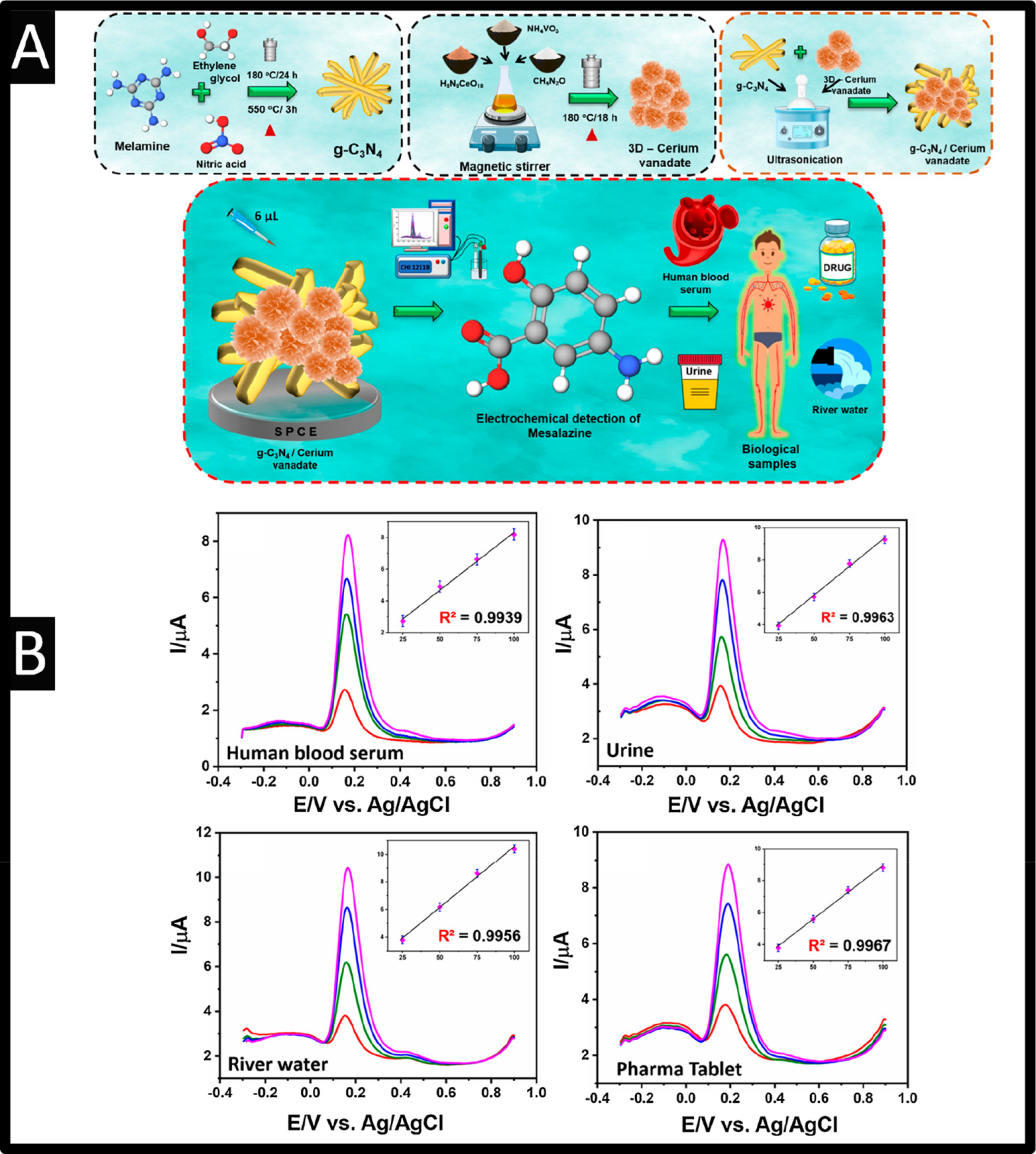
(A) Schematic diagram for the use of CeVO_4_ modified graphitic-carbon nitride; (B) differential pulse voltammetry for standard additions into the various samples; Reproduced from ref (45). Copyright 2021 Elsevier.

In another approach, rare-earth metal vanadate (CeVO_4_) has been used with graphitic-carbon nitride (g-C_3_N_4_) nanotubes immobilized upon SPCEs;^45^ please see Figure 5A. The g-C_3_N_4_ was fabricated through taking melamine, and ethylene glycol with nitric acid which is stirred for 1 h at a temperature of 90 °C, after which, the solution is autoclaved via heating for 24 h at 180 °C. TEM images confirmed that the nanotubes were in the range of 50 nm. Next, the 3D cerium vanadate was fabricated by taking vanadate and cerium salts dissolved in deionized water and adding urea slowly, followed by stirring for 3 h at 50 °C; this was then transferred into the Teflon hydrothermal autoclave for 18 h at 120 °C. The average diameters of CeVO_4_ nanostructures are in the range of ∼1–2 μM consisting of nanopetals (sheet-layers), which are interconnected one to another while they comprise several thin-layers of CeVO_4_ with an average thickness of ∼15–20 nm. Last, the g-C_3_N_4_ was mixed with the 3D cerium vanadate via ultrasound which was then loaded onto a SPCEs. This sensor was shown to exhibit a linear range of 2 nM to 380 μM and LOD of 5.47 nM.^45^ The sensor was shown to be resilient to the 100-fold concentration of different potential interfering species: azathioprine, sulfasalazine, l-alanine, ascorbic acid, glucose, sucrose, Cl^–^, K+, and Na+, where only a change of 5% of the current was observed. This sensor was shown to be successfully applied for the sensing of 5-ASA within human blood serum and urine, river water, and a pharmaceutical tablet; where the standard additions can be observed via Figure 5B The authors note as the sensor is fabricated with SPCE are simple, low cost with high sensing abilities which has shown that 5-ASA has been shown to be useful within biological, water, and pharmaceutical samples opening a wide range of feasibility in future.^45^

Other notable work that has reported a low LOD of 0.8 nM with a linear range of 3 nM to 350 μM used carbon aerogels supported on Pd–WO_3_ nanorods (CAs/Pd–WO_3_) hybrid nanocomposite via sol–gel and microwave-assisted methodology.^46^ The carbon aerogels are modified with WO_3_ nanorods via a microwave-assisted synthesis which are then deposited upon SPCE. TEM analysis of the palladium nanoparticles revealed that the average size of 10 nm. Chronoamperometry responses are reported where the response for 5-ASA additions can be observed along with the stability which was run up to 4000 s. Furthermore, the response of interfering species where the effect of the main serum and urine components interfering species, 1 mM of creatinine, bilirubin, human serum albumin, -uric acid, and common interfering species, 0.5 mM of acetamiprid, riboflavin, 4-nitrophenol, catechol, and hydroquinone are shown. The authors note that their results revealed that the subsequent injections of serum and urine components (creatinine, bilirubin, human serum albumin, uric acid) show a slight increase in electrochemical sensor response. However, all these common interfering compounds had no interference. This sensor was applied to the measurement of 5-ASA within human urine and blood serum samples where a drop of acetonitrile, 1:1 is mixed to precipitate blood and urine components which results in recoveries from 99.6–100.4%. The response of the sensor is reported to be due to highly active nanointerfaces upon the porous carbon aerogels surfaces which results in exhibiting its superior electrochemical oxidative activity to detect 5-ASA.^46^

## 5 Boron-Doped Diamond Electrodes (BDDEs)

Comparing BDDEs with noble metals and glassy carbon, BDDEs have the advantages of having a wider electrochemical potential window for aqueous (∼3–3.5 V) and nonaqueous media (∼5.0–7.5 V), a small yet stable background current where the electrochemical properties tunable by the boron concentration in the diamond lattice, presence of sp^2^ carbon, and surface termination, and last but yet important, is that the sp^3^ hybridized structure of BDDEs makes it resistant to biofouling and biocompatible with organisms; please consult ref (47) for an elegant overview of using BDDEs. Surprisingly, there is only one report using a BDDE for the sensing of 5-ASA.^48^ Of note is that the electrochemical oxidation peak occurs at +900 mV while taking into account the slight deviation used in the reference electrodes, this peak has an significant overpotential than that reported using GCE.^23^ The author reported that the sensor can measure 5-ASA over the range of 2 μM – 0.3 mM with a LoD of 0.02 μM. In exploring their sensor, they measure interferents noting that sucrose, glucose, urea, and barbituric acid did not affect the voltammetric peak, but others resulted in a slight decrease of the 5-ASA signal ascorbic acid, creatinine but notably folic acid interfered at low concentrations, while at high concentrations it completely overlapped the signal of 5-ASA which could not be evaluated correctly—please see later that 5-ASA can be simultaneously measured using a CPEs. We note that unfortunately, folic acid and folates, respectively, are abundant in the organisms and within biological samples—this limits its real use in measuring 5-ASA of patients.

## 6 Carbon (Graphite) Paste Electrodes (CPEs)

**Figure 6:**
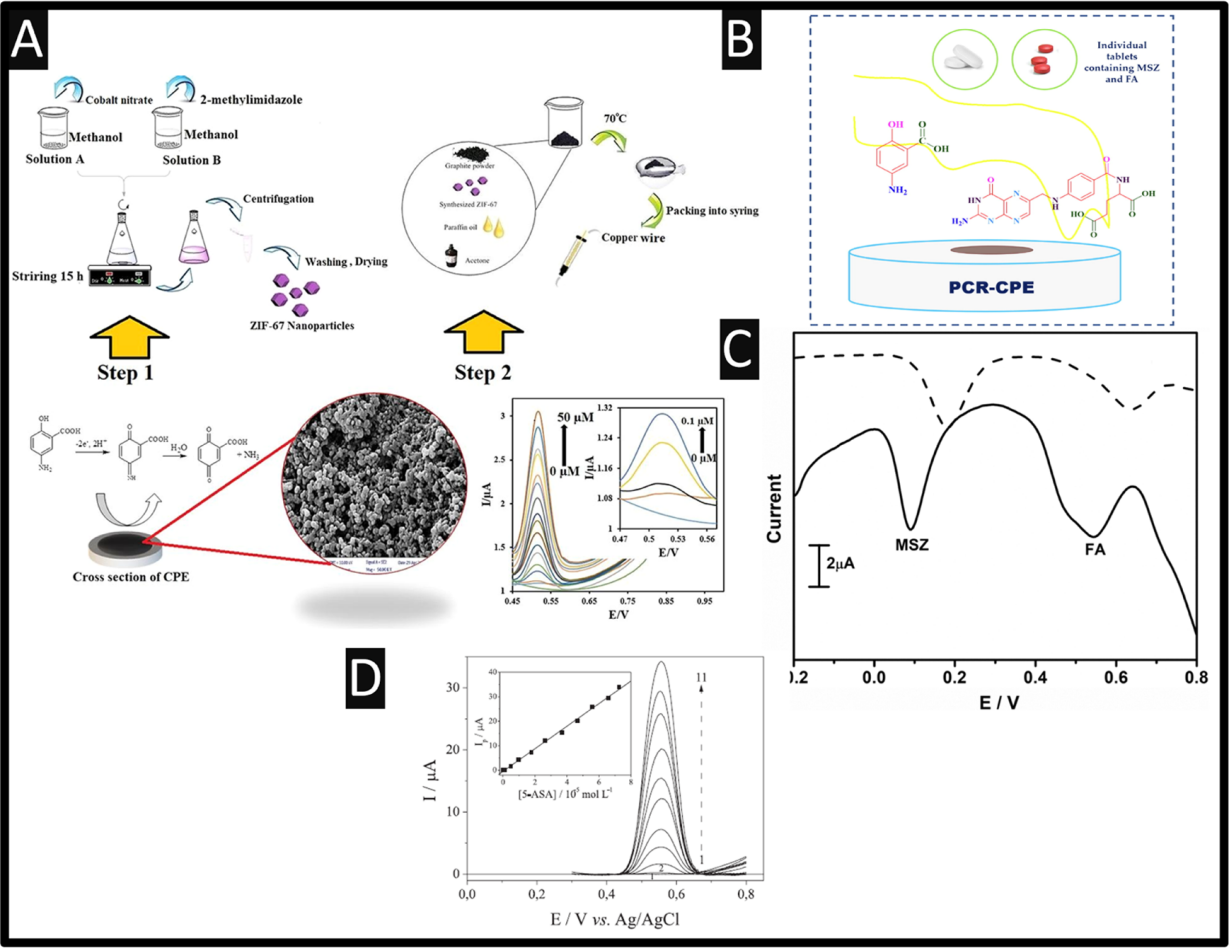
(A) Summary of how the authors they fabricated ZIF-67, how they incorporated this into a CPEs and a typical calibration experiment. Reproduced from ref (56). Copyright 2020 Elsevier. (B) An overview of the authors polymerized-congo red modified CPEs; (C) simultaneous detection of folic acid and 5-ASA where the dotted line is the absence of polymerized-congo red while the solid line shows the response of the polymerized-congo red CPEs. Reproduced from ref (57). Copyright 2022 Elsevier.

CPEs are easily fabricated using powdered carbon (graphite) which is mixed with a binder, such as paraffin oil or nujol mull^49^ to hold the material together. CPEs are useful since they are nontoxic, possess chemical inertness, low ohmic resistance, renewability, robustness, where in the case of problems with surface passivation via interferents, these are eliminated by a simple and quick renewal of their surface; please consult references^50,51^ for a thorough overview. CPEs have been used in the determination of 5-ASA which has reported a poly(benzoquinone) chromium(III) complex was fabricated via a one pot and facile methodology.^52^ This complex is mixed into the bulk of the CPEs which gives rise to a peak at +0.6 V (vs. Ag/AgCl) which was compared to a blank CPE. The current increase is presumed to be due to the presence of chromium(III) and the quinone functional groups.^52^ The authors optimized the pH response toward 5-ASA reporting that a pH of 2 gave rise to the largest electroanalytical signal, which they explored their electrodes to additions of 5-ASA over the range 2–600 μM reporting a low LoD of 70 nM. The author determined the concentration of 5-ASA within a pharmaceutical tablet which they compared with an indirect spectroscopy approach with good agreement, while reporting their electroanalytical sensor has recoveries from 99 to 120%. Other work using CPEs have reported the use of a modified electrode surface with the surfactant sodium dodecyl sulfate toward 5-ASA.^53^ The authors report that in the presence of the surfactant that the signal is greater compared to a blank electrode surface which is attributed to the negative charge from the surface reacting with the positively charged 5-ASA. The authors reported a narrow linear range of 1–7 μM with a LoD of 0.238 μM and they demonstrated that their sensor is useful in measuring 5-ASA within pharmaceutical tablets. This work has been extending using the cetyltrimethylammonium bromide surfactant,^54^ and the use of hexadecyltrimethylammonium bromide supported on a nanocomposite of carbon dots deposited onto a GCE.^55^ Other work has reported on Zeolitic imidazole frameworks (ZIF-67) nanoparticles mixed into the bulk of a CPEs.^56^ An overview of how they fabricated the ZIF-67, Figure 6A, involves a solvothermal methodology where cobalt salt is dissolved into methanol and polyvinylpyrrolidone followed by stirring for 15 h. This solution is added to 2-methylimidazole dissolved in methanol, which are mixed and transferred into a autoclave where temperatures are set at 170 °C for 4 h. The temperature is reduced to 120 °C for 12 h and then finally dropped to 80 °C for 24 h, which results in a purple solid; the distribution of the particle size is 30 nm. The ZIF-67 particles are incorporated into the bulk of CPEs which demonstrated a linear range of 0.03–50 μM with a LoD of 0.01 μM. This sensor was applied to the measurement of 5-ASA within human urine and blood serum samples where in the case of the urine it was diluted with 1 mL of urine to 9 mL of a buffer (pH 2), while in the case of the blood serum samples, they are transferred into a centrifugal filtration tube which a 10-fold dilution is applied. The spiked samples achieved 96–104% recoveries.

Lastly, we summarize work published by Ganesh et al.^57^ who have developed a sensor involving polymerized-congo red modified CPEs; see Figure 6B, where the modification gives rise to voltammetric peaks from polymerized-congo red while the signal of 5-ASA is well resolved. This sensor is able to measure 5-ASA over the range of 80–200 μM with a LoD reported to be 0.112 μM which they applied to its measurement with pharmaceutical tablets. Of note, this sensor was applied to the measurement of 5-ASA and folic acid; please see Figure 6C. Folic acid is used as a water-soluble vitamin for biological function of cell metabolisms, and this can appear within medical and pharmaceutical samples. To this end, as shown within Figure 6C, there are two distinctive peaks which have a separation of 550 mV. The authors studied the effect of interferents within a 10-fold access of sucrose, ammonium chloride, dextrose, potassium oxalate, calcium sulfate, calcium chloride, and sodium chloride which did not make any change to the signal of the 5-ASA; the authors demonstrated that the sensor can measure 5-ASA within a pharmaceutical tablet.^57^

## 7 Pencil Graphite Electrode

The use of pencil graphite electrodes is another form of carbon electrode platforms used for electroanalytical applications which is from commercially available graphite pencil leads, are low cost, low background current, and ease of renewal.^59^ Pencil graphite leads are composed of graphite, 65%, clay, 30% and a binder, typically wax, resins of polymer based.^59^ Uliana and co-workers have used a pencil graphite electrode for the sensing of 5-ASA which provided a linear range of 0.978–72.5 μM with a LoD of 0.02 μM.^58^ The authors did not study any effect from interferent but turned to the sensing of 5-ASA within a pharmaceutical tablet which was compared with independent HPLC which confirmed no difference between the proposed electroanalytical sensor with HPLC. Other work using a pencil graphite electrode have used a deoxyribonucleic acid (DNA) biosensor using a polypyrrole/sponge-like carbon/La^3+^-doped CuO (PPy/SL-C/La^3+^-doped CuO) nanocomposite for the sensing of 5-ASA. The La^3+^-doped CuO nanocomposite was synthesized by taking lanthanum and copper metal salts which are dissolved into ethanol and a ammonia solution. Then they added in sodium hydroxide and sodium nitrate which they heated at 100 °C for 24 h resulting in the formation of the nanocomposite. The unique structure, sponge like, has a thickness of 18–21 nm. This nanostructure is carbonized by heating at 600 °C within an argon oven resulting in carbon/La^3+^-doped CuO nanocomposites. Pyrrole is added into a phosphate buffer solution with carbon/La^3+^-doped CuO nanocomposites which are electropolymerized using cyclic voltammetry from −0.2 to +1.0 V for 4 cycles at 50 mVs^–1^ (vs Ag/AgCl). Lastly, ds-DNA was added via immobilization through holding an electrochemical potential of +0.5 V (vs. Ag/AgCl) for 270 s. This nanocomposite was evaluated to addition of 5-ASA over the range of 0.03–100 μM with a LoD of 9 nM.^60^ The author explored the selectivity of the sensor through exploring 100-fold concentration of glucose, norepinephrine, ascorbic acid, citrate, folic acid, and 200-fold concentration of calcium, sodium, and chloride which showed no effect. The analytical determination of 5-ASA was evaluated within spiked human urine and serum and a pharmaceutical tablet where they observed recoveries from 97% to 104%, while the authors note that they need to undertake future work exploring this with LC-MS.

## 8 Comparison to Analytical Approaches

As shown within Table 2, we have compared the electroanalytical sensing platforms with those of other analytical instruments which show that they provide similar dynamic ranges with associated LoDs and are all applied to real sample composition. The use of electroanalytical sensors for the detection of 5-ASA provides significant advantage over analytical instrumentation since these are inexpensive, rapid, sensitive, selective, and can be miniaturized to be given to healthcare providers to measure 5-ASA quickly and on-site. Furthermore, in some cases using electroanalytical approaches allow us to effectively bypass the derivatization, solvent extraction, and centrifugation steps that are common to analytical instruments,^16^ where the pre-electroanalytical step for human plasma protein precipitation was added into nonaqueous solvents.^61^

**Table 2:** Comparison of the Various Analytical Reports for the 5-ASA Determination

determination methodology	dynamic range	limit of detection	real sample composition	reference
high-performance liquid chromatography with fluorescence	1 μM–1 mM	0.02 μM	human plasma	(10)
liquid chromatography/positive-ion electrospray ionization mass spectrometry	0.065–6.53 μM	0.065 μM	human plasma	(12)
high-performance liquid chromatography/electrospray ionization tandem mass spectrometry	0.32–26.1 μM	0.098 μM	human plasma	(13)
liquid chromatography with fluorescence	0.6–52 μM	0.13 μM	human plasma and urine	(13)
electrochemical	50 nM–2.5 μM	12 nM	human serum	(15)
electrochemical	0.03–100 μM	9 nM	human urine, serum and pharmaceutical tablet	(60)
electrochemical	1 nM–500 μM	2.9 nM	human serum, urine and pharmaceutical	(33)

## 9 Summary and Outlook

We have summarized the various approaches for the electroanalytical sensing of 5-ASA which shows that there are many different approaches that can measure the target analytes at the micro and nano levels, utilizing the advantages in the development of nanomaterials. Future work needs to utilize SPCEs as these can bridge the gap from laboratory experiments to supporting commercialization. Nanomaterial modified SPCEs can provide in situ sensing platforms to be used by healthcare providers, where due to the fabrication and scales-of-economy a single measurement can be provided without the need to clean the electrode surface as is required for GCE, BDD etc. Other work needs to emphasize the use of comparing the electroanalytical response with spiked real samples with that of classical laboratory analytical instrumentation to ensure that it is validated, encouraging commercialization. What is evident is that everyone measures 5-ASA within pharmaceutical tablets and spiked human urine, serum, and river water, but in the context of serum, there is only one paper that considers the sensing of 5-ASA and its derivative.^15^ It is reported that 5-ASA is metabolized by *N*-acetyltransferase into *N*-acetyled-5-ASA derivative in the liver and intestinal tract.^15^ This compound is the major metabolite present in blood with a half-life of up to 10 h while in plasma, both 5-ASA and *N*-acetyled-5-ASA are found 40–50% and 80%, respectively bound to proteins;^61,62^ the measurement of both are vital to evaluate the pharmacokinetics of 5-ASA. Clearly, further research needs to measure both 5-ASA and *N*-acetyled-5-ASA via electroanalytical approaches. Furthermore, no one has yet used electroanalytical sensing in real measurements, i.e., collecting plasma from volunteers after a single-dose oral administration of 5-ASA in order to measure both 5-ASA and *N*-acetyled-5-ASA and evaluate the pharmacokinetics, pharmacodynamics, and efficacy of the drug^39^—further work needs to address this.
